# Behavioral Responses of Wild Rodents to Owl Calls in an Austral Temperate Forest

**DOI:** 10.3390/ani11020428

**Published:** 2021-02-07

**Authors:** Mᵃ Carmen Hernández, Denise M. Jara-Stapfer, Ana Muñoz, Cristian Bonacic, Isabel Barja, André V. Rubio

**Affiliations:** 1 Laboratory of Etho-Physiology, Department Biology (Unit Zoology), Universidad Autónoma de Madrid, C/Darwin 2, Campus Universitario de Cantoblanco, 28049 Madrid, Spain; mariacarmen.hernandez@uam.es (M.C.H.); isabel.barja@uam.es (I.B.); 2 Departamento de Ciencias Biológicas Animales, Facultad de Ciencias Veterinarias y Pecuarias, Universidad de Chile, Santa Rosa 11735, La Pintana, Santiago 8820808, Chile; denise.jara.st@gmail.com; 3 Departamento de Ecosistemas y Medio Ambiente, Facultad de Agronomía e Ingeniería Forestal, Pontificia Universidad Católica de Chile, Santiago 8940000, Chile; asmunoz1@uc.cl (A.M.); bona@uc.cl (C.B.); 4 Escuela de Medicina Veterinaria, Pontificia Universidad Católica de Chile, Santiago 8940000, Chile; 5 Research Centre in Biodiversity and Global Change (CIBC-UAM), Universidad Autónoma de Madrid, 28049 Madrid, Spain

**Keywords:** landscape of fear, predation risk, predator–prey interactions, small mammals

## Abstract

**Simple Summary:**

Growing human populations are challenging scientists to find effective ways to control and mitigate human–wildlife conflict while preserving biodiversity. It has been reported that predator odor and calls can drive away rodents, but little is known about species-specific responses of prey. For these reasons, we compared the behavioral changes of common rodent species inhabiting the Chilean temperate forest (*Abrothrix* spp., the long-tailed pygmy rice rat *Oligoryzomys* *longicaudatus* and the black rat *Rattus rattus*) when exposed to two different native predator calls (the austral pygmy owl *Glaucidium nana* and the rufous-legged owl *Strix rufipes*) and a control (no predator calls). Our results showed that all rodent species modified their behavior in the presence of predator calls, but the effects were species dependent. These findings point to the need to carefully study target rodent species instead of applying a general control plan for all rodent species.

**Abstract:**

Ecologically based rodent management strategies are arising as a sustainable approach to rodent control, allowing us to preserve biodiversity while safeguarding human economic activities. Despite predator signals being known to generally repel rodents, few field-based studies have compared the behavioral effects of several predators on different prey species, especially in Neotropical ecosystems. Here, we used camera traps to study the behavior of rodent species native to the Chilean temperate forest (*Abrothrix* spp., long-tailed pygmy rice rat *Oligoryzomys longicaudatus*) and an introduced rodent (black rat *Rattus rattus*). Using playbacks of raptor calls, we experimentally exposed rodents to three predation risk treatments: austral pygmy owl calls (*Glaucidium nana*), rufous-legged owl calls (*Strix rufipes*) and a control treatment (absence of owl calls). We evaluated the effects of the treatments on the time allocated to three behaviors: feeding time, locomotor activity and vigilance. Moonlight and vegetation cover were also considered in the analyses, as they can modify perceived predation risk. Results showed that predator calls and environmental factors modified prey behavior depending not only on the predator species, but also on the rodent species. Consequently, owl playbacks could be regarded as a promising rodent control tool, knowing that future studies would be critical to deeply understand differences between species in order to select the most effective predator cues.

## 1. Introduction

Rodents are the most diverse order of mammals [1], and a small portion of them (~7–10%) are considered agricultural and infrastructural pests [2], as well as important reservoirs of a variety of zoonotic diseases [3,4]. Therefore, rodent management strategies are needed in order to prevent and control rodent populations in a wide range of human-dominated landscapes [5].

Because rodents are prey to several predators (reptiles, birds and mammals), they can perceive risk of predation in response to auditory, visual, and olfactory cues emanating from their predators [6,7,8,9]. Recently, the use of perceived predation risk has been proposed as a management tool for ecologically based rodent management, under The Landscape of Fear (LOF) framework [10,11,12], in which rodent populations could be managed by manipulating their perceived predation risk to deter them from target areas [11]. Therefore, the presence of a rodent predator can be simulated by using direct cues, such as carnivore urine and feces or raptor calls [12,13,14], inducing antipredator behaviors, such as modifications to space-use, foraging activity, social behavior and vigilance patterns [13,14,15,16].

Despite chemosensory cues being the most common signals used by small mammals to assess predation risk [17], the importance of auditory cues should not be overlooked, as they can provide equally valuable information [12]. In rodents, several laboratory and field experiments using playbacks of raptor calls successfully triggered antipredator responses in wild rodents [12,18,19,20].

In southern Chile, small rodents are commonly found in agroecosystems and peridomestic settings [21,22]. Some of these rodents are reservoirs of several zoonotic pathogens, including the Andes virus (ANDV), which causes hantavirus cardiopulmonary syndrome (HCPS) in humans [23]. The main reservoir of ANDV is the long-tailed pygmy rice rat (*Oligoryzomys longicaudatus*) [24]. Furthermore, serologic evidence of ANDV has also been found to a lesser extent in other native species such as the long–haired mouse (*Abrothrix hirta*) and the olive grass mouse (*A. olivacea*), and even in invasive species such as *Rattus* spp. [22,25]. Since HCPS and other rodent-borne zoonoses are a major concern for public health, there is a need to better understand the behavioral ecology of zoonotic hosts to find effective solutions to prevent human exposure to rodents and their pathogens.

The rodent species mentioned above are common prey to diverse raptors, such as the Austral pigmy owl (*Glaucidium nana*) and the Rufous-legged owl (*Strix rufipes*) [26]. *Glaucidium nana* is a small habitat generalist raptor (body length ~ 200 mm) that hunts and/or nests within forests, shrublands, and around human settlements [27]. Small mammals are important components of its diet, preying upon a wide variety of rodents, including *A. hirta*, *A. olivaceus*, *O. longicaudatus* [26,28]. *Strix rufipes* is a medium-sized forest specialist owl (body length ~ 400 mm) that hunts and nests only within forests [27]. It is a generalist feeder, but it preys mostly on small mammals and insects. The most consumed small mammal preys are scansorial and arboreal species, such as *O. longicaudatus*, the arboreal rat (*Irenomys tarsalis*) and the colocolo opossum (*Dromiciops gliroides*) [27,29]. Hence, playback calls of these species may be expected to induce antipredator responses in these rodents. However, to our knowledge, this topic has not been addressed.

For these reasons, the main objective of this study was to assess how direct auditory predation risk cues (i.e., owl calls) affect the behavior (feeding, vigilance and movement patterns) of a rodent assemblage composed of native species (*Abrothrix* spp. and *O. longicaudatus*) and an introduced species (*Rattus rattus*) inhabiting a temperate forest in southern Chile. We also wanted to test if there were response differences depending on the predator species (*G. nana* vs. *S. rufipes*), shrub cover thickness and moonlight (indirect cues of predation risk). Therefore, we tested the following predictions: (a) Rodents would increase vigilance and decrease total feeding time under risk of predation. In addition, rodent movement patterns would be affected by the presence of predator calls, limiting locomotion in higher risk settings. (b) Rodent behavioral responses would be stronger when moonlight is greater or shrub cover is scarce, due to a higher perceived risk. (c) Rodents would modulate their antipredator responses depending on the owl species because predator dietary preferences pose different perceived risks to each rodent species. (d) Native rodents would exhibit a more finely tuned response depending on the predator selected, as they have been exposed to natural selection driven by these predators, while *R. rattus* was introduced in the past centuries. The exact date of introduction of *R. rattus* to Chile is unknown, but likely dates back to the mid-1600s [30], and to date it has colonized a wide range of natural areas [31].

## 2. Materials and Methods

### 2.1. Study Area and Target Species

The study was conducted in a temperate forest located in Huelemolle, at the Villarrica lake basin (39°16′ S, 71°48′ W) in the Araucanía Region (southern Chile). The climate in this area is temperate-humid with a short dry season (<4 months) in summer (January–March) and an average yearly rainfall of 2000 mm [32]. Mean minimum and maximum temperatures are, respectively, 10.4 °C and 25.3 °C in the warmest month (January) and 4.2 °C and 12.1 °C in the coldest month (July) [32]. Forests are dominated by Patagonian oak (*Lophozonia obliqua*) and coigue (*Nothofagus dombeyi*), mainly associated with Chilean laurel (*Laurelia sempervirens*), olivillo (*Aextoxicon punctatum*), ulmo (*Eucryphia cordifolia*) and lingue (*Persea lingue*) [33]. Several raptors inhabit the temperate forest of southern Chile [31]. The most abundant owls in the study area are *G. nana* and *S. rufipes* (Ibarra, data unpublished). Other predators of rodents commonly found in this area are foxes (*Lycalopex griseus*, *L. culpaeus*) and the kodkod cat (*Leopardus guigna*) [34].

To determine the composition of the rodent community in the study area, we performed a live-trapping survey in six plots during April 2020. Each plot consisted of 42 Sherman live traps shaping a 6 × 7 grid set at 5 m intervals. Each plot was sampled during nine consecutive nights (trapping effort = 2268 traps/night). A total of 123 individuals were captured. Rodent assemblage was composed of four species: *A. hirta*, *A. olivaceus*, *O. longicaudatus* (native species) and *R. rattus* (exotic species). Both *Abrothrix* species are terrestrial and omnivorous. *A. hirta* (until 2014 considered a synonym of *A. longipilis*) is a medium-sized rodent (body length~130 mm), while *A. olivaceus* is smaller (body length~90 mm). *O. longicaudatus* is small (~90 mm), scansorial (i.e., ability or propensity to climb), and omnivorous. *R. rattus* is a large species (~200 mm), scansorial and omnivorous.

### 2.2. Field Methods

We conducted an experimental field study during the austral autumn (May 2019), when several rodent species reach their highest abundances in the year. The experiment had three treatments, two of which simulated the presence of owls (predation risk treatments): (1) *G. nana* calls, and (2) *S. rufipes* calls. The third was the control treatment (without owl vocalizations). For each treatment, we installed two grids (2 grids × 3 treatments = 6 grids), each one consisting of four foraging stations separated by 25 m (Figure S1), similar to other studies on rodent predation risks, in terms of distance between stations and grid configuration [8]. Therefore, each treatment had a total of eight foraging stations. To avoid overlapping owl calls between treatments, the distance between grids was at least 300 m. This distance was tested in the study area, and smaller distances (e.g., 200 m) have been used in other studies in temperate forests [35]. All grids were similar in rodent abundance (mean = 20 individuals) and composition among them, according to the live-trapping survey described above. Each grid in the experiment was sampled for three consecutive nights, simultaneously sampling one grid per treatment (Figure S2) to reduce possible temporal/environmental variations between days. Each grid was only used for one treatment type. We did not implement enclosures or a semi-captive experiment, therefore, all foraging stations could be visited by rodents around the area.

Each foraging station contained 30 g of rolled oats with vanilla extract placed on a plastic petri dish, located on the forest ground. An infrared motion-triggered camera trap (Bushnell Trophy Cam, 119537C, Bushnell Optics, Overland Park, Kansas) was installed at each foraging station to record videos of the rodents visiting the station. Each camera was mounted horizontally 1.5 m above the ground on a PVC pipe [36]. Cameras were set to take 30-sec videos once an individual visited a foraging station and continued recording as long as at least one individual stayed in front of the sensor (0 sec intervals between videos) [37]. Cameras were activated from dusk (6 pm) to dawn (7 am), since rodents in Chilean temperate forests are mostly nocturnal [38], and set for 3 consecutive nights at each foraging station. Each camera recorded an area of 0.89 m^2^. The medium sensitivity setting was applied to all cameras, and two layers of masking tape were added to the cameras to reduce flash brightness for close-range operation. Camera traps have been used to monitor small mammals, as they can readily detect and reliably identify small mammals to species [36,37]. Before the experiment, we conducted pilot trials of camera performance in the field to test their effectiveness in recording rodent presence and distinguish individual behaviors.

We also estimated vegetation cover of shrubs by averaging ocular estimates of shrub cover to the nearest 5% within a square frame of 1 m^2^ centered at each foraging station [39]. Each 1 m^2^ frame is divided into 10 cm × 10 cm cells, creating proportional compartments to estimate shrub coverage. Moon phase (%, where full moon = 100%) for each night surveyed was also registered (https://www.timeanddate.com/moon/phases/chile/villarrica (accessed on 6 February 2020)). These variables are commonly used as indirect cues of predation risk.

### 2.3. Vocalizations and Playbacks

To simulate the presence of owls, playback stimuli were broadcasted in both predation risk treatments (*G. nana* calls and *S. rufipes* calls). We used the most common owl calls available in recordings; in the case of *G. nana*, we used the three most common types of vocalizations: mating call or partner contact, aggression call and juvenile vocalization. In the case of *S. rufipes*, two types of vocalizations were emitted: a location call, used to delimit territory and maintain couple ties, and a territorial call. The sound system (JBL Charge 3 speaker and mp3 player, Sony NWZ-B183F (Tokyo, Japan)) was set up on a tree (2 m height) at the center of each grid (12.5 m apart from foraging stations). From dusk to dawn, playbacks were emitted randomly, alternating periods of one to three min, with silence periods of one to five min [40].

### 2.4. Behavioral Analysis

We analyzed the following rodent behaviors in the recorded videos: (1) feeding time: the total amount of time spent foraging at the stations; (2) fast locomotor activity: total amount of time spent running; (3) slow locomotor activity: total amount of time spent walking; (4) vigilance: total amount of time spent standing still, with head and ears pricked, looking around. These behaviors are usually used in studies of perceived predation risk [40,41].

Video recordings did not allow reliable identification of specific individuals. Therefore, all analyses were to the species level. For rodents of the genus *Abrothrix*, it was difficult to identify both species in all recordings, therefore we combined their records as *Abrothrix* spp. At each foraging station, we organized video recordings by “events” to distinguish between independent detections of each species. An event was considered as a video or several videos of the same species within a five-minute interval [42]. Therefore, we grouped behavioral analyses for each event, and we summed the time dedicated to each behavior within each event. When more than one individual was recorded in the same video, we included the behavior of each individual as a different event.

### 2.5. Statistical Analysis

Behavioral responses to owl calls were analyzed using Generalized Linear Models (GLMs) with Gamma distribution and Log as the link function, since data did not fit a normal distribution. We performed three different GLMs, one for each rodent species (*Abrothrix* spp., *O. longicaudatus*, and *R. rattus*), considering the following response variables: feeding time, locomotor activity and vigilance. The predictor variables were: predation risk treatment (control/*G. nana*/*S. rufipes*), moon phase, and vegetation cover at each foraging station, considering the four stations in each grid as pseudoreplicates. Moreover, we set the total number of videos of each event as the offset to make data comparable and to avoid any possible bias caused by small-scale variations in rodent population densities. Results were considered significant at α < 0.05. Data are represented as mean ± standard error (SE). The software used to perform the statistical analysis was SPSS 23.0 for Windows (SPSS Inc., Chicago, IL, USA).

### 2.6. Ethics Statement

Rodent sampling was authorized by Servicio Agrícola y Ganadero (SAG; Chilean Fish and Wildlife Service) under permit No. 7479/2018. Bioethical approval (No. 18197-VET-UCH) was issued by the Faculty of Animal and Veterinary Sciences, University of Chile.

## 3. Results

The total number of events analyzed in this study was 981, corresponding to 2253 different video recordings. Numbers of events by rodent species were 551 for *Abrothrix* spp., 182 for *O. longicaudatus* and 248 for *R. rattus*.

### 3.1. Behavioral Responses of Abrothrix Spp.

**Figure 1:**
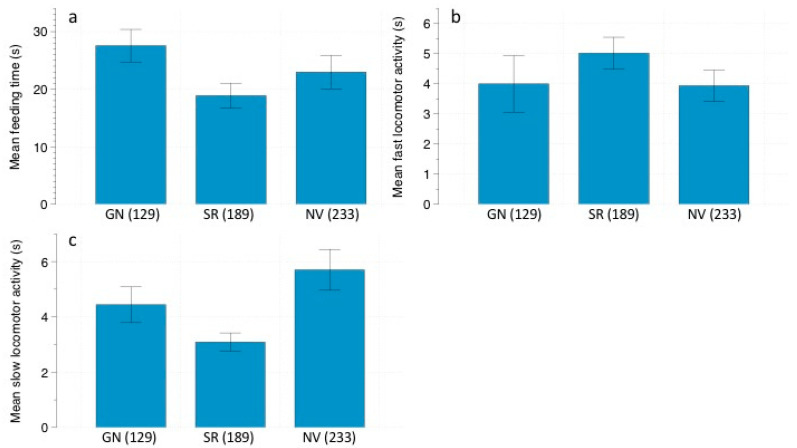
(**a**) Mean feeding time (s) ± SE, (**b**) mean fast locomotor activity (s) ± SE, and (**c**) mean slow locomotor activity (s) ± SE displayed by *Abrothrix* spp. depending on predation risk: *G. nana* calls (GN)/*S. rufipes* calls (SR)/No vocalizations (NV). Number of events are in parenthesis.

**Figure 2:**
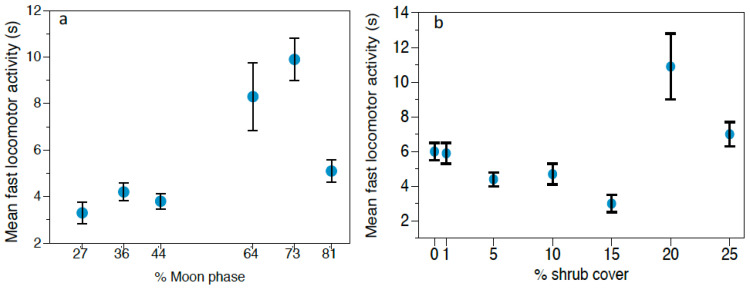

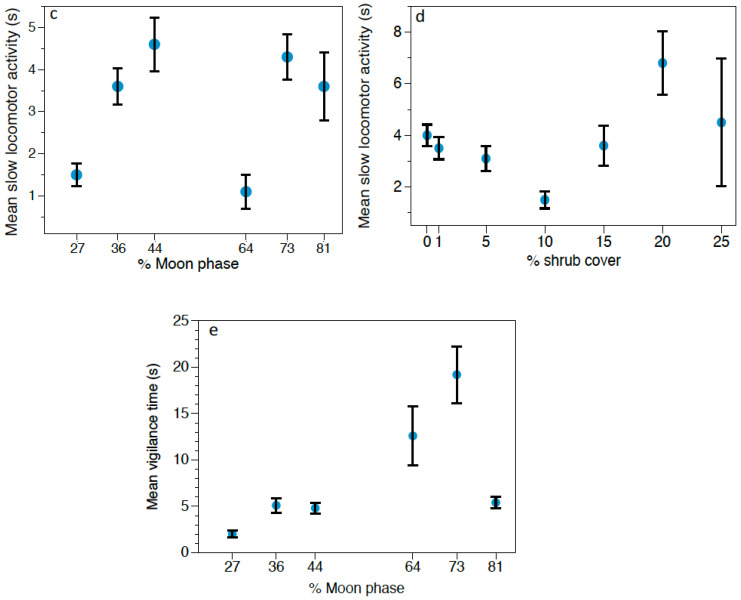
*Abrothrix* spp. behaviors. Mean fast locomotor activity (s) ± SE depending on (**a**) moonlight and (**b**) shrub cover thickness. Mean slow locomotor activity (s) ± SE depending on (**c**) moonlight and (**d**) shrub cover thickness. Mean vigilance time (s) ± SE depending on moonlight (**e**).

We found that owl calls modulated *Abrothrix* spp. feeding behavior (Table 1). Individuals spent significantly more time feeding in the control treatment (22.25 ± 3.20 s; N = 233), compared to the *G. nana* (19.09 ± 1.88 s; N = 129) and *S. rufipes* treatments (17.86 ± 2.46 s; N = 189) (Figure 1a). For fast locomotor activity, moonlight and shrub cover had a statistically significant influence on *Abrothrix* spp. swift movements (Table 2). Moonlight and dense bush cover were linked to an increase in *Abrothrix* spp. fast movements (Figure 2a,b). Regarding the slow locomotor activity response, this variable was modulated by moonlight, shrub cover and predation risk treatment (Table 3). A bimodal distribution was found for moonlight (Figure 2c), with *Abrothrix* spp. allocating more time to slow movements when shrub cover was thicker (Figure 2d) and during the *G. nana* treatment (5.27 ± 0.61 s; N = 129 vs. 3.312 ± 0.36 s for *S. rufipes* and 3.14 ± 0.31 s for the control) (Figure 1c). On the other hand, *Abrothrix* spp. vigilance behavior appeared to be only significantly affected by moonlight, being less vigilant when moonlight was less intense (Table 4 and Figure 2e).

### 3.2. Behavioral Responses of Oligoryzomys longicaudatus

**Figure 3:**
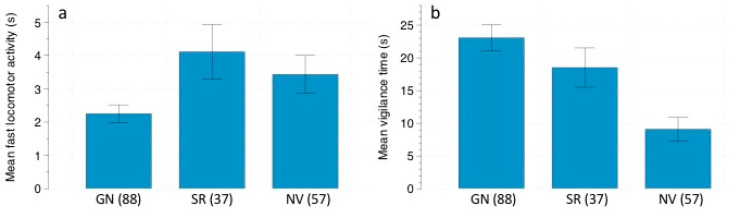
(**a**) Mean fast locomotor activity (s), and (**b**) mean vigilance time (s) ± SE displayed by *O. longicaudatus* depending on predation risk: *G. nana* calls (GN)/*S. rufipes* calls (SR)/No vocalizations (NV). Number of events are in parenthesis.

**Figure 4:**
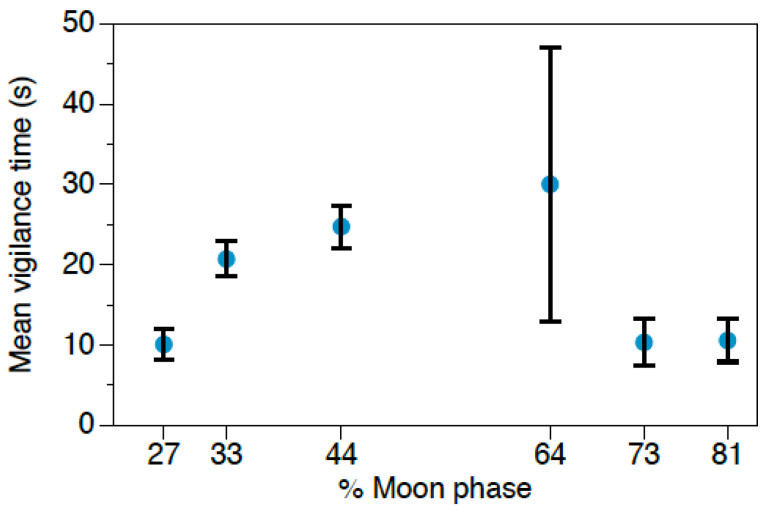
Mean vigilance time (s) ± SE displayed by *O. longicaudatus* depending on moonlight.

Total feeding time exhibited by *O. longicaudatus* did not differ among treatments (Table 5; omnibus test F = 3.33; df = 4; *p* = 0.504). Overall fast locomotor activity of *O. longicaudatus* was significantly modulated by owl call treatments (Table 6). Individuals were more active during the *S. rupifes* treatment (4.11 ± 0.82 s; N = 37) compared to the *G. nana* treatment (2.25 ± 0.27 s; N = 88) and the control (3.43 ± 0.57 s; N = 57) (Figure 3a). The GLM analyzing the effects on slow locomotor activity was not statistically significant (Table 7; omnibus test F = 9.224; df = 4; *p* = 0.056). Vigilance behavior was significantly affected by predation risk treatment and moonlight (Table 8). Mean total time per event allocated to vigilance was significantly lower in the control (9.08 ± 1.83 s; N = 57) compared to the *G. nana* (23.05 ± 2.00 s; N = 88) and *S. rufipes* treatments (18.51 ± 3.00 s; N = 37) (Figure 3b). Regarding moonlight, mice increased their vigilance when moonlight reached intermediate levels (Figure 4).

### 3.3. Behavioral Responses of Rattus rattus

**Figure 5:**
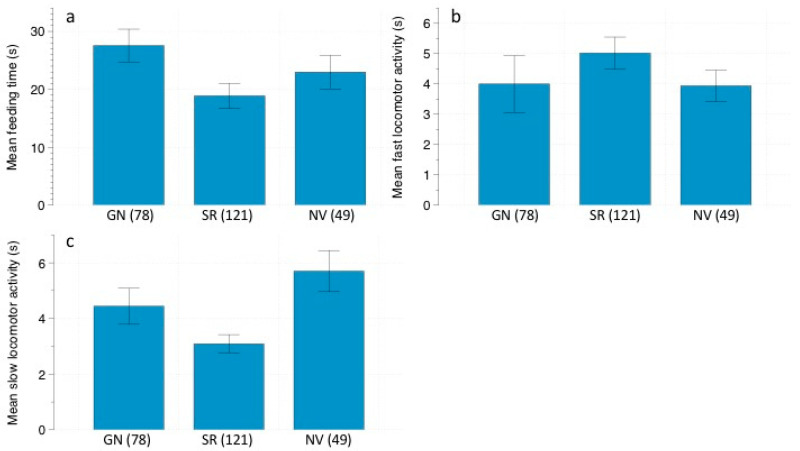
(**a**) Mean feeding time (s) ± SE, (**b**) mean fast locomotor activity (s) ± SE, and (**c**) mean slow locomotor activity (s) ± SE, displayed by *R. rattus*, depending on predation risk: *G. nana* calls (GN)/*S. rufipes* calls (SR)/No vocalizations (NV). Number of events are in parenthesis.

**Figure 6:**
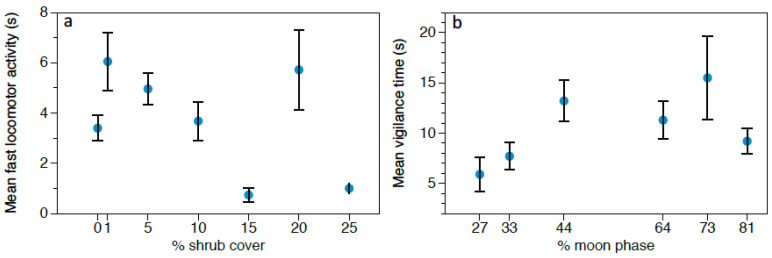
(**a**) Mean fast locomotor activity time (s) ± SE displayed by *R. rattus* depending on the shrub cover thickness. (**b**) Mean vigilance time (s) ± SE of *R. rattus* depending on moonlight.

The only variable that affected the time spent in feeding behavior was predation risk treatment (Table 9). *R. rattus* allocated more time to feeding in the *G. nana* treatment (27.56 ± 2.83 s; N = 78), followed by the control (22.98 ± 2.89 s; N = 49), and lastly, the *S. rufipes* treatment (18.88 ± 2.18 s; N = 121) (Figure 5a). Regarding fast locomotor activity, we found that predation risk treatment and shrub cover affected this behavior (Table 10). Fast movements increased during the *S. rufipes* treatment (5.02 ± 0.53 s; N = 121) compared to the *G. nana* treatment (4.00 ± 0.94 s; N = 78) and the control (3.94 ± 0.52 s; N = 49) (Figure 5b). Regarding shrub cover, we found a bimodal distribution, with one peak at lower shrub cover densities and the other at greater shrub thickness (Figure 6a). As for the effect on slow locomotor activity, we found that only predation risk treatment significantly affected this behavior (Table 11). Slow movements were significantly greater in the control (5.71 ± 0.744 s; N = 49) compared to the owl treatments (*G. nana* 4.44 ± 0.65 s; N = 78; *S. rufipes* 3.09 ± 0.33 s; N = 121) (Figure 5c). Finally, vigilance increased with moonlight (Table 12). A bimodal distribution of this response was found again, reaching one peak at intermediate moonlight levels and the other when moonlight was more intense. (Figure 6b).

## 4. Discussion

In accordance with our predictions, our findings showed that owl calls of both raptors and environmental factors (moonlight and shrub cover) had influences on rodent behavior, suggesting that playbacks of owl vocalizations can induce antipredator responses in all rodent species analyzed, and that response differences are species dependent. Contrary to our predictions, we found that the introduced *R. rattus* had a marked antipredator response to native owl calls, akin to native rodents.

### 4.1. Abrothrix Spp.

The recorded decrease in time spent feeding by *Abrothrix* spp. in the presence of owl calls was consistent with the predation risk allocation hypothesis proposed by Lima & Bednekoff [43]. The temporal variation in risk would cause prey species to focus on fitness-enhancing activities when perceived predation risk is low, whereas in riskier settings, animals should display the highest antipredator efforts, diverting time from the rest of daily activities [16,44,45]. Regarding vigilance behavior, our results would also be in accordance with the predation risk allocation hypothesis, as *Abrothrix* spp. decreased the time allocated to this antipredatory behavior when moonlight was less intense [43,46,47]. In addition, vocalizations of *G. nana* prompted *Abrothrix* spp. to increase their slow movements, which could be linked to the predator inspection phenomenon [48,49].

The increase in fast movements when moonlight was more intense suggests that *Abrothrix* spp.’s perceived predation risk was higher [50], meaning that individuals displayed antipredator responses (i.e., fleeing behavior) when detectability was enhanced by nocturnal light. Furthermore, *Abrothrix* spp. spent more time on slow locomotor activity during nights with moonlight of high and low-medium intensity, which may be explained by the trade-off between searching for food and safety and the different antipredator strategies exhibited by individuals [10,51,52]. With regard to shrub cover, *Abrothrix* spp. increased both fast and slow movements when shrub cover was thicker, which may indicate that vegetation cover makes individuals feel safer [53,54]. However, vegetation cover could not only be linked to predation risk, but also to other non-considered factors which could have influenced this response. For example, denser patches could provide better thermic isolation for individuals, affecting their thermoregulation and energy budget, which could impact the energy available for locomotion [55,56]. Nevertheless, we should take these results with caution, as our analysis was limited to the camera coverage area, and movement patterns over a larger area could also be important to analyze.

### 4.2. Oligoryzomys longicaudatus

*O. longicaudatus* has phenotypic features (e.g., jumping ability, partial bipedalism) that give it a better chance of escaping from predator attacks [57,58]. Therefore, this rodent can explore riskier patches without needing to change its foraging behavior [57]. These features might explain the lack of effect of owl calls and indirect cues (moonlight and shrub cover) on feeding time. On the contrary, this rodent triggered other antipredator responses, increasing vigilance behavior in both raptor treatments and also increasing fast locomotor activity, particularly during the *S. rufipes* treatment. The latter suggests that *O. longicaudatus* opts for swift fleeing behavior instead of freezing in response to owl calls.

Moonlight had an effect on *O. longicaudatus* vigilance behavior, for which we found that the time spent on this conduct was greater when moonlight exhibited intermediate levels. This result could suggest that the importance of vigilance behavior is crucial in settings where luminosity is sufficient for mice to still rely on visual clues, but not too high so as to expose them to predator detection. On the other hand, no effect of shrub cover was found. This might be because *O. longicaudatus* can use patches with less shrub cover compared to other Chilean rodents [59], as its partial bipedalism provides faster escape responses.

### 4.3. Rattus rattus

Our findings indicate that *S. rufipes* would be a more dangerous predator to *R. rattus* than G. nana, as these rodents allocated less time to feeding in the *S. rufipes* treatment, as well as increased their fast movements and decreased their slow movements. This assumption finds empirical support in the fact that *S. rufipes* preys on larger rodents (including *R. rattus*) than *G. nana* [60,61]. Furthermore, *G. nana* is a small owl (~21 cm length, 60–95 g weight; [26]), smaller and lighter than *R. rattus*, which can make hunting *R. rattus* difficult [62].

These results suggest *R. rattus* is well-adapted to recognize and modulate its behavior in response to native predator calls. A previous study in Australia described that *R. rattus* can recognize evolutionarily familiar dogs and foxes, but is naïve towards Australian native species such as quolls (*Dasyurus maculatus*) [63]. Contrary to our findings, that study reported that *R. rattus* did not alter its behavior in the presence of carnivore odors. A possible explanation could be that owl calls are unequivocal signals of the immediate presence of the predator, while chemical cues can persist in the environment when the predator has long dispersed. Another explanation is that *R. rattus* was introduced to Australia 150 years ago, while this rodent was introduced more than 300 years ago to Chile, and therefore has had more time to adapt to native predators.

On the other hand, we found two bimodal distributions in the models analyzing *R. rattus* fast locomotor response in relation to shrub cover and vigilance behavior depending on moonlight. These bimodal patterns could indicate that individual factors, such as sex, age, breeding condition, personality or previous experience cause two different responses to predation risk [64,65]. These individual factors entail different energetic demands for individuals, probably causing diversification in antipredatory responses to maximize fitness [10]. Further studies would be needed to find which factors could be modulating this response and to disentangle its effects.

### 4.4. Final Remarks and Future Directions

This work is a first step in understanding the antipredator behavior of rodents in the Chilean temperate forests, and our findings could be particularly interesting for future ecologically based rodent management strategies. Our results pointed out that strategies based on the landscape of fear should carefully consider which predator would be more effective depending on the target rodent. Moreover, understanding prey ecology and how they react to changes in key environmental factors (e.g., vegetation cover and moonlight) would be critical to concentrate predator treatments in settings where the prey has a particularly low perceived risk [11].

We acknowledge that this study has limitations that should be considered. First, the experiment consisted of a small number of foraging stations, which may limit the statistical power of the results. Also, distance between foraging stations within each sampling plot does not guarantee absolute independence among them, although similar distances have been used in other studies regarding small rodents and predation risk [8,10,16]. Therefore, future studies should include a greater number of sampling sites, which would allow an increase in replicates and a greater number of individuals. Second, this study was limited to behaviors in front of the camera that covered a small area frame. Although this methodology has been increasingly used in studies of rodents and their behaviors [16,37,65], this small area frame may limit some of the behavioral analysis, mainly regarding movement behaviors.

For further studies, we also suggest combining field and laboratory experiments that can help to better understand rodent responses to predator cues, since during field experiments animals are usually exposed to other environmental variables that may influence animal behaviors that are difficult to control. Future studies should focus on the long-term effects of exposure to predator calls, evaluating the impact of habituation in the efficacy of this rodent management strategy [66]. Moreover, it could also be interesting to study the joint effect of predator calls from different species acting simultaneously, since a particular combination could reach higher effectiveness in decreasing rodent activity [7]. Lastly, understanding how rodent responses vary locally and temporally also requires further research. Rodents may behave differently in urban, rural and well-preserved native habitats [67], and seasonality may also be relevant to changes in rodent behavior [68,69].

## 5. Conclusions

This work was the first study reporting behavioral responses of rodents to owl calls in temperate forests in Chile. Our study provides empirical evidence that perceived predation risk is a species-dependent phenomenon that can vary not only depending on the predator species involved, but also on the prey species within a rodent assemblage. Since these rodents can also be found in peridomestic habitats and are reservoir hosts of several zoonoses, further studies using playbacks of owl calls should be conducted in order to generate scientific knowledge for planning ecologically based rodent management strategies within the landscape of fear framework.

## Figures and Tables

**Table 1:** Results of the GLM analyzing the effect of experimental and environmental factors on *Abrothrix* spp. feeding behavior.

Effect	*F*	df	*p*
Intercept	25.49	1	0.000
Predation risk treatment	14.54	2	0.001
Moonlight	0.09	1	0.767
Shrub cover	0.03	1	0.859

**Table 2:** Results of the GLM analyzing the effect of experimental and environmental factors on *Abrothrix* spp. fast locomotor activity.

Effect	*F*	df	*p*
Intercept	0.61	1	0.435
Predation risk treatment	17.68	2	0.274
Moonlight	16.78	1	0.001
Shrub cover	7.01	1	0.035

**Table 3:** Results of the GLM analyzing the effect of experimental and environmental factors on *Abrothrix* spp. slow locomotor activity.

Effect	*F*	df	*p*
Intercept	9.85	1	0.002
Predation risk treatment	21.91	2	0.000
Moonlight	21.18	1	0.000
Shrub cover	5.96	1	0.015

**Table 4:** Results of the GLM analyzing the effect of experimental and environmental factors on *Abrothrix* spp. vigilance behavior.

Effect	*F*	df	*p*
Intercept	5.55	1	0.019
Predation risk treatment	1.50	2	0.471
Moonlight	5.54	1	0.019
Shrub cover	1.36	1	0.243

**Table 5:** Results of the GLM analyzing the effect of experimental and environmental factors on *O. longicaudatus* feeding behavior.

Effect	*F*	df	*p*
Intercept	51.02	1	0.000
Predation risk treatment	0.09	2	0.956
Moonlight	0.48	1	0.489
Shrub cover	2.48	1	0.115

**Table 6:** Results of the GLM analyzing the effect of experimental and environmental factors on *O. longicaudatus* fast locomotor activity.

Effect	*F*	df	*p*
Intercept	0.43	1	0.513
Predation risk treatment	19.11	2	0.000
Moonlight	2.12	1	0.146
Shrub cover	3.37	1	0.066

**Table 7:** Results of the GLM analyzing the effect of experimental and environmental factors on *O. longicaudatus* slow locomotor activity.

Effect	*F*	df	*p*
Intercept	1.422	1	0.233
Predation risk treatment	6.607	2	0.037
Moonlight	9.22	1	0.337
Bush cover	0.069	1	0.792

**Table 8:** Results of the GLM analyzing the effect of experimental and environmental factors on *O. longicaudatus* vigilance behavior.

Effect	*F*	df	*p*
Intercept	72.86	1	0.000
Predation risk treatment	11.86	2	0.003
Moonlight	14.44	1	0.000
Shrub cover	1.98	1	0.160

**Table 9:** Results of the GLM analyzing the effect of experimental and environmental factors on *R. rattus* feeding behavior.

Effect	*F*	df	*p*
Intercept	43.51	1	0.000
Predation risk treatment	12.30	2	0.002
Moonlight	1.62	1	0.203
Shrub cover	0.44	1	0.505

**Table 10:** Results of the GLM analyzing the effect of experimental and environmental factors on *R. rattus* fast locomotor activity.

Effect	*F*	df	*p*
Intercept	0.14	1	0.713
Predation risk treatment	15.28	2	0.000
Moonlight	2.57	1	0.109
Shrub cover	15.20	1	0.000

**Table 11:** Results of the GLM analyzing the effect of experimental and environmental factors on *R. rattus* slow locomotor activity.

Effect	*F*	df	*p*
Intercept	0.49	1	0.486
Predation risk treatment	8.77	2	0.012
Moonlight	1.57	1	0.210
Shrub cover	0.64	1	0.424

**Table 12:** Results of the GLM analyzing the effect of experimental and environmental factors on *R. rattus* vigilance behavior.

Effect	*F*	df	*p*
Intercept	20.96	1	0.000
Predation risk treatment	1.73	2	0.421
Moonlight	9.29	1	0.002
Shrub cover	0.69	1	0.407

## Supplementary Materials

The following are available online at https://www.mdpi.com/2076-2615/11/2/428/s1, Figure S1: Diagrams of grids and foraging stations. Figure S2: Timeline of the experiment.
