# Supplementary material for: Behavioral Responses of Wild Rodents to Owl Calls in an Austral Temperate Forest
Source: Animals (Basel). 2021 Feb 7;11(2):428. doi: 10.3390/ani11020428 (PMC7916001; doi:10.3390/ani11020428)
Supplement: Supplementary file 1 [file animals-11-00428-s001.pdf]

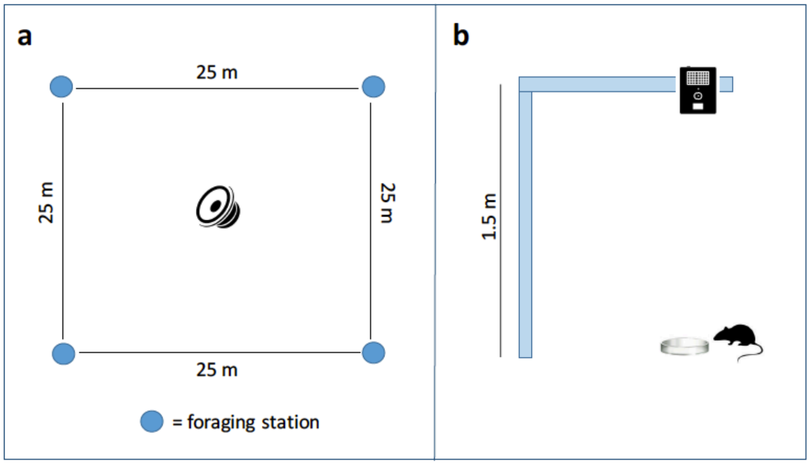

Figure S1. Diagrams of grids and foraging station. (a) Diagram of an experimental grid. (b) Diagram of a foraging station.
For each treatment, we installed two grids (2 grids x 3 treatments = 6 grids), each one consisting in four foraging stations separated by 25 m. Therefore, each treatment had a total of eight foraging stations.
Each foraging station contained 30 g of rolled oats with vanilla extract placed on a plastic petri dish, located on the forest ground. An infrared motion-triggered camera trap (Bushnell Trophy Cam, 119537C, Bushnell Optics, Overland Park, Kansas) was installed at each foraging station to record videos of the rodents visiting the station. Each camera was mounted horizontally 1.5 m above the ground on a PVC pole.

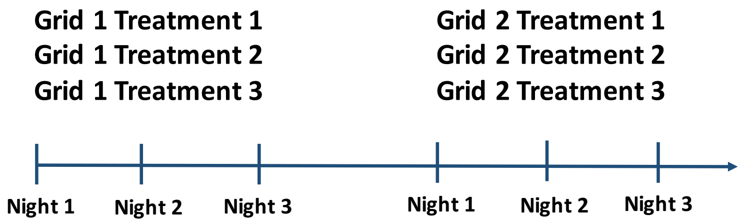

Figure S2. Timeline of the experiment. At each grid, the experiment lasted three consecutive nights and one grid per treatment were sampled simultaneously to reduce possible temporal/environmental variations between days. All grids were used only for one treatment type. The experiment was conducted from May 23th 2019 to May 29th 2019.
